# A Personalized Task Allocation Strategy in Mobile Crowdsensing for Minimizing Total Cost

**DOI:** 10.3390/s22072751

**Published:** 2022-04-02

**Authors:** Hengfei Gao, Hongwei Zhao

**Affiliations:** College of Computer Science and Technology, Jilin University, Changchun 130012, China; gaohf16@mails.jlu.edu.cn

**Keywords:** mobile crowdsensing, personalized task allocation, minimizing cost, traveling salesman problem

## Abstract

Mobile crowdsensing utilizes the devices of a group of users to cooperatively perform some sensing tasks, where finding the perfect allocation from tasks to users is commonly crucial to guarantee task completion efficiency. However, existing works usually assume a static task allocation by sorting the cost of users to complete the tasks, where the cost is measured by the expense of time or distance. In this paper, we argue that the task allocation process is actually a dynamic combinational optimization problem because the previous allocated task will influence the initial state of the user to finish the next task, and the user’s preference will also influence the actual cost. To this end, we propose a personalized task allocation strategy for minimizing total cost, where the cost for a user to finish a task is measured by both the moving distance and the user’s preference for the task, then instead of statically allocating the tasks, the allocation problem is formulated as a heterogeneous, asymmetric, multiple traveling salesman problem (TSP). Furthermore, we transform the multiple-TSP to the single-TSP by proving the equivalency, and two solutions are presented to solve the single-TSP. One is a greedy algorithm, which is proved to have a bound to the optimal solution. The other is a genetic algorithm, which spends more calculation time while achieving a lower total cost. Finally, we have conducted a number of simulations based on three widely-used real-world traces: roma/taxi, epfl, and geolife. The simulation results could match the results of theoretical analysis.

## 1. Introduction

With the explosive usage of smartphones and the widely equipping of powerful sensors on them, a practical offline crowdsourcing scheme called Mobile CrowdSensing (MCS) [1] becomes popular in our daily life over the past few years, which recruits a group of users to commonly finish some location-based sensing tasks through their hand-held devices. A traditional MCS system [2,3,4,5,6,7,8,9,10] has three roles: a centralized platform, task publishers, and mobile users. The platform takes charge of addressing the requestings from task publishers and announcing the corresponding sensing tasks to mobile users as a form of notification in their mobile-device applications.

A common challenge in crowdsening is to find a suitable allocation from tasks to users, in order to achieve an optimal task completion. To this end, most of the existing researches [11,12,13,14,15,16,17] regard task allocation as a static matching problem between users and tasks. In most cases, they first measure the contribution of a user to all the tasks, then the ranking of contributions is regarded as important references to select suitable users. However, we argue that the task allocation process is a dynamic combinational optimization problem because the previous assigned task will influence the initial location of the user to head for the next task. Hence, we should consider the problem as a continuous and dynamic allocation process. Moreover, existing works usually consider time or distance spent for moving to a task location as the actual cost, while ignoring the user’s preference for the task. Actually, the user’s preference may make a discount for the time or distance cost. For example, we suppose such a task, which is located at a shopping mall, and a user likes shopping very much. Then even though the mall is far away from the user, the user may still be willing to complete the task. Obviously, the above two limitations could be further improved to enhance the efficiency of task allocation.

**Figure 1:**
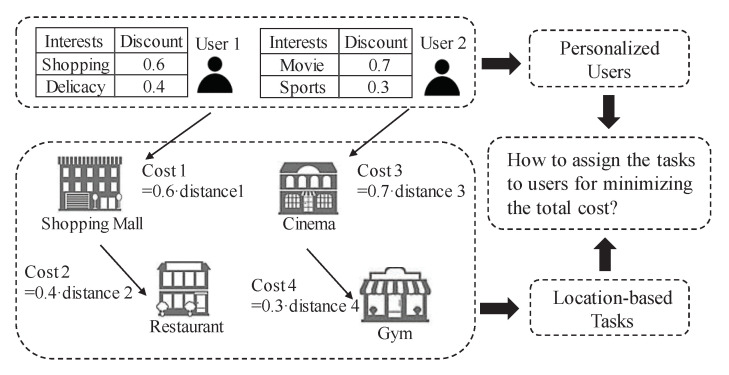
Personalized task allocation strategy for mobile crowdsensing. The cost for a user to finish a sensing task depends on not only the distance but also the user’s interest on the location of task. Then the problem is transformed into how to assign the tasks to users for minimizing the total cost.

Considering the above limitations of existing task allocation researches, in this paper, we design a Personalized Task Allocation strategy in Mobile crowdsensing (PTAM), with the purpose of minimizing the total cost for the users to complete the sensing tasks. As shown in Figure 1, the cost for a user to finish a task depends on not only the distance but also the user’s interests. Then for user 1, the cost (Cost 2) from shopping mall to restaurant does not only equal to the distance, the interests of user may achieve a discount for the actual cost. Obviously, there are two roles in Figure 1: personalized users, and location-based tasks. The problem turns into how to assign tasks to users for minimizing the total cost of completing all the tasks and getting back to the initial locations of users.

In order to solve the above problem, we first formulate the problem as a heterogeneous, asymmetric, multiple TSP. Then, we transform the multiple-TSP to single-TSP, which could be solved through both greedy and genetic algorithms. Furthermore, we make the greedy algorithm with an acceptable bound to the optimal solution, and also make the genetic algorithm with heuristic close to the optimal solution. The above research thoughts raise the following challenges: (1) due to the reason that the estimated cost takes the user’s preferences into consideration, then the costs do not satisfy geometric property. Hence, the formulated TSP is a heterogeneous, asymmetric, multiple TSP problem; (2) the simplest TSP is NP-hard, while the formulated problem in this paper is much more complex than the traditional TSP; (3) different from the cost maximization problem, the cost minimization problem in TSP could not be directly solved by a bounded greedy algorithm.

The main contributions of this paper are briefly summarized as follows:

- A cost estimation method is proposed by taking the user’s preference for the sensing task into consideration. Furthermore, the minimizing cost problem is formulated as solving a heterogeneous, asymmetric, multiple TSP.
- Through transforming multiple-TSP to single-TSP, we first propose a greedy algorithm: PTAM-Greedy when the task is urgent, which is proved to have a bound to the optimal solution.
- When the task is not urgent, we further propose a genetic algorithm mixed with heuristic: PTAM-Genetic to minimize the total cost. The genetic algorithm consumes a lot of calculation time while achieving a better total cost performance.
- We conduct a number of simulations based on three widely-used real-world traces. The simulation results show that, PTAM-Greedy achieves a bounded cost performance, and PTAM-Genetic achieves the lowest total cost compared with the other task allocation strategies.

The remainder of the paper is organized as follows. The system model and problem formulation are presented in Section 2. The personalized task allocation strategies (PTAM-Greedy and PTAM-Genetic) are detailedly described in Section 3. In Section 4, we evaluate the performance of the task allocation strategies proposed in this paper by conducting a number of simulations. The related works are introduced in Section 5. Finally, we conclude the paper in Section 6.

## 2. System Overview

### 2.1. System Model

We consider a MCS system including a set of mobile users, denoted by $U=\{u_{1},u_{2},\cdots ,u_{n}\}$ and also a set of tasks: $S=\{s_{1},s_{2},\cdots ,s_{m}\}$. At the system beginning time, each user has an initial location, and all the tasks also have their corresponding locations. Moreover, all the users’ preferences are denoted by the set $A=\{a_{1},a_{2},\cdots ,a_{r}\}$. Without loss of generality, $u_{i}$’s preferences are $A_{u_{i}}\subseteq A$, while each task location $s_{i}$ could meet some preferences of users, which are $A_{s_{i}}\subseteq A$. The physical distance between $u_{i}$ and $s_{p}$ is recorded as $D(u_{i},s_{p})$, and the distance between $s_{p}$ and $s_{q}$ is $D(s_{p},s_{q})$. Accordingly, $C^{i}\left(u_{i},s_{p}\right)$ represents the cost for user *i* to finish sensing task $s_{p}$ from its initial location. While $C^{i}\left(s_{p},s_{q}\right)$ represents the cost for user *i* to finish sensing task $s_{q}$ from its previous task location $s_{p}$. As described before, the cost *C* depends on not only the distance *D*, but also the user’s preference *A*.

Each $u_{i}$ begins with its initial location, and heads for its first task location $s_{p}$ with the cost $C^{i}\left(u_{i},s_{p}\right)$. In the following steps, if $u_{i}$ is at the location of $s_{p}$, its cost to finish the next task $s_{q}$ is $C^{i}\left(s_{p},s_{q}\right)$. We assume that a task $s_{p}$ could be finished by a user who arrives at the location of $s_{p}$. In other words, we do not consider the data sensing and uploading process. Moreover, all the tasks should be allocated to at least one user, if a task sequence is assigned to a user, the user needs to begin with its initial location and head for the locations of task sequence one by one, and finally go back to its initial location. For example, if the task sequence $\left\{s_{1},s_{3}\right\}$ is allocated to $u_{1}$, then $u_{1}$ will consume the cost $C^{1}\left(u_{1},s_{1}\right)+C^{1}\left(s_{1},s_{3}\right)+C^{1}\left(s_{3},u_{1}\right)$ to finish the tasks. In this way, the locations of users and tasks are regarded as nodes, and the costs are considered as edges with weight among nodes. If we determine the allocation from tasks to users, a unidirectional weighted topological graph consisting of cycles is formulated. The notations used throughout this paper are listed in Table 1.

### 2.2. Problem Description

By regarding the users and tasks as nodes, and considering the corresponding costs as edges, we get a unidirectional weighted topological graph. We attempt to assign the tasks to the users, and also determine the order in which the tasks are completed. In other words, we want to allocate the tasks to users in the manner of task sequences. If a task sequence is assigned to a user, then the user should complete the tasks one by one following the sequence order. A task allocation strategy is composed of $S_{i}$ and $C_{i}$, where $S_{i}$ is the task set allocated to $u_{i}$, while $C_{i}$ is the total cost for $u_{i}$ to complete task sequence and get back to initial location. Hence, our purpose is to find the best task allocation meeting the following optimal problem:

(1) $$\begin{array}{cc} \; & \mathrm{Minimize}\;\sum_{i=1}^{n}C_{i}\\ \; & s.t.\;\;\forall s\in S,\;\exists S_{i},\;s\in S_{i} \end{array}$$

Here, we aim to find the best task allocation to minimize the total cost for all the users, with the constraint that each task is at least assigned to one user. It is worth noting that, maybe some users are not assigned with any task, while all the tasks should be allocated. If necessary, a task may be assigned to multiple users.

## 3. Personalized Task Allocation Strategy

**Figure 2:**
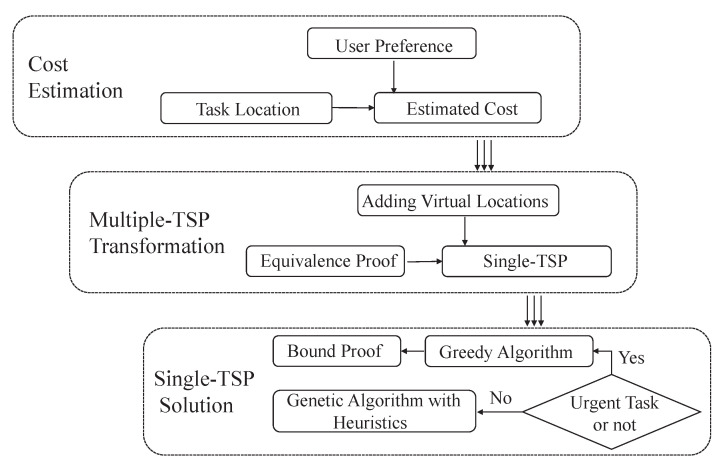
The task allocation system framework.

In this section, we detailedly describe all the modules in task allocation system framework as shown in Figure 2. It mainly includes the following three parts: cost estimation, which estimates the actual costs for the edges among nodes in the unidirectional weighted topological graph; multiple-TSP transformation, which transforms the formulated multiple-TSP to a single-TSP; and single-TSP solution, which solves the transformed single-TSP by both greedy and genetic algorithms.

### 3.1. Cost Estimation and Multiple-TSP Formulation

First, we focus on the calculation process for the weights of edges among nodes in the formulated unidirectional graph. As previous described, the cost for $u_{i}$ to move from $s_{p}$ to $s_{q}$ is $C^{i}\left(s_{p},s_{q}\right)$, which mainly depends on not only the distance between $s_{p}$ and $s_{q}$: $D(s_{p},s_{q})$, but also $u_{i}$’s preference for $s_{q}$. Obviously, a longer distance $D(s_{p},s_{q})$ should lead to a higher cost because $u_{i}$ needs to move a long distance to finish the task. While if $u_{i}$ is interested in the location of task $s_{q}$, then the actual cost should have a discount because $u_{i}$ perhaps would like to head for its interested task location even though the location is far away from $u_{i}$. Hence, we should give a reasonable estimated cost which considers not only the distance between the user and task location but also the user’s preference for the task.

In order to solve the above problem, the key is to measure the $u_{i}$’s preference level for $s_{q}$. We adopt the tag-matching method to measure the preference level, which means that we mark both user’s preferences $A_{u_{i}}$ and task location’s attributes $A_{s_{q}}$ from a common attribute set *A*. Then the first step is to measure the $u_{i}$’s preference level $x_{iq}$ for task $s_{q}$. We use the following equation to calculate $x_{iq}$:

(2) $$\begin{array}{c} x_{iq}=\frac{|A_{u_{i}}⋂A_{s_{q}}|}{|A_{u_{i}}|} \end{array}$$

Obviously, xisin[0,1], if a user’s preference could match all the tags of location $s_{q}$, then x=1. Otherwise, x<1. Then, we attempt to calculate the discount for $u_{i}$ to task $s_{q}$, which is defined as $d_{iq}$:

(3) $$\begin{array}{c} d_{iq}=\left(d_{max}-1\right)\sqrt{1-{\left(1-x_{iq}\right)}^{2}}+1, \end{array}$$

where $d_{max}$ is a constant ($0<d_{max}<1$), which represents the maximum discount. Obviously, if $x_{iq}=1$, which means $u_{i}$ is totally interested in $s_{q}$, then $d_{iq}=d_{max}$. It is not difficult to find that when $x_{iq}=0$, $d_{iq}=1$, this is because if $u_{i}$ is totally not interested in $s_{q}$, then there is no discount for the actual cost. Moreover, $d_{iq}$ is an decreasing function of $x_{iq}$: $\frac{\partial d_{iq}}{\partial x_{iq}}<0$, this is because a larger interest leads to a better discount. While this function is convergent: $\frac{\partial^{2}d_{iq}}{\partial {x_{iq}}^{2}}>0$. The above descriptions also explain that why we use Equation (3) as the discount function.

Finally, the actual cost of $u_{i}$ to move from the location of $s_{p}$ to the location of $s_{q}$ is defined as the Equation (4). Obviously, $d_{max}D\left(s_{p},s_{q}\right)⩽C^{i}\left(s_{p},s_{q}\right)⩽D(s_{p},s_{q})$, and the value of $C^{i}\left(s_{p},s_{q}\right)$ depends on not only the distance but also the preference.

(4) $$\begin{array}{c} C^{i}\left(s_{p},s_{q}\right)=d_{iq}D\left(s_{p},s_{q}\right) \end{array}$$

After calculating the weights of edges, we now focus on the structure of formulated unidirectional graph. It is not difficult to find that, the cost $C^{i}\left(s_{p},s_{q}\right)$ may be different from $C^{i}\left(s_{q},s_{p}\right)$. Hence, the formulated unidirectional graph is asymmetrical. Moreover, for different users, the costs of them to move from $s_{p}$ to $s_{q}$ may be also different. So the formulated unidirectional graph is heterogeneous. To sum up, the problem changes to be finding the optimal task allocation (assigning task sequences to users) to minimize the total cost (depends on not only the distance between user and task but also the user’s preference for the task) in the unidirectional, heterogeneous and asymmetrical weighted graph. It is not difficult to find that, in fact, this is equivalent to solving a heterogeneous and asymmetrical multiple-TSP [18].

### 3.2. Transformation from Multiple-TSP to Single-TSP

**Figure 3:**
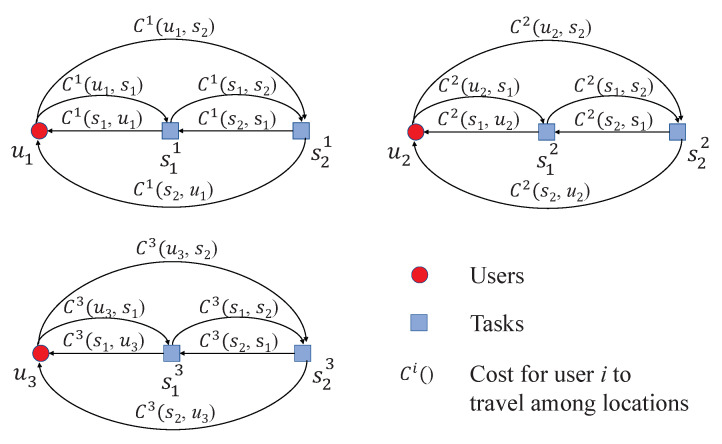
An example of the virtual task locations and costs for 3 users and 2 task locations.

In order to solve the multiple-TSP, we transform it to the equivalent single-TSP [19]. The detailed process of the transformation is described as follows. First, we replicate a set of virtual task locations for each user. The virtual task locations for user *i* is defined as $s_{j}^{i},\forall j\in \left\{1,\ldots ,m\right\}$. For each i∈1,…,n, $s_{j}^{i}$ is the virtual task location of $s_{j}$ for user *i*. The cost of moving from location $s_{p}^{i}$ to $s_{q}^{i}$ of user *i* is denoted by $C^{i}\left(s_{p},s_{q}\right)$ for all p,q∈{1,…,m}. As shown in Figure 3, each user has a replicated virtual task location corresponding to each physical task location.

Then, we add a virtual terminal point for each user. Specifically, we denote $u_{i}^{v}$ as the terminal point of user *i*. So there are m+2 nodes corresponding to user *i*. Due to the fact that there are *n* users, the total number of nodes in the transformed graph is n(m+2). The costs of the edges on the transformed graph are calculated as follows:

(5) $$\begin{array}{cc} C(u_{i},s_{j}^{i})=\; & C^{i}\left(u_{i},s_{j}\right)+B,\forall i\in \left\{1,\ldots ,n\right\},\\ \; & \forall j\in \{1,\ldots ,m\}.\\ C(u_{i},u_{i}^{v})=\; & B,\forall i\in \{1,\ldots ,n\}.\\ C(u_{i}^{v},u_{i+1})=\; & 0,\forall i\in \{1,\ldots ,n-1\}.\\ C(u_{n}^{v},u_{1})=\; & 0.\\ C(s_{p}^{i},s_{q}^{i+1})=\; & C^{i+1}\left(s_{p},s_{q}\right)+B,\forall i\in \left\{1,\ldots ,n-1\right\},\\ \; & \forall p,q\in \{1,\ldots ,m\},p\neq q.\\ C(s_{p}^{n},s_{q}^{1})=\; & C^{1}\left(s_{p},s_{q}\right)+B,\forall p,q\in \left\{1,\ldots ,m\right\},p\neq q.\\ C(s_{j}^{i},s_{j}^{i+1})=\; & 0,\forall i\in \{1,\ldots ,n-1\},\forall j\in \{1,\ldots ,m\}.\\ C(s_{j}^{n},s_{j}^{1})=\; & 0,\;\forall j\in \{1,\ldots ,m\}.\\ C(s_{j}^{i},u_{i+1}^{v})=\; & C^{i+1}\left(s_{j},u_{i+1}\right)+B,\forall i\in \left\{1,\ldots ,n-1\right\},\\ \; & \forall j\in \{1,\ldots ,m\}.\\ C(s_{j}^{n},u_{1}^{v})=\; & C^{1}\left(s_{j},u_{1}\right)+B,\forall j\in \left\{1,\ldots ,m\right\}. \end{array}$$

**Figure 4:**
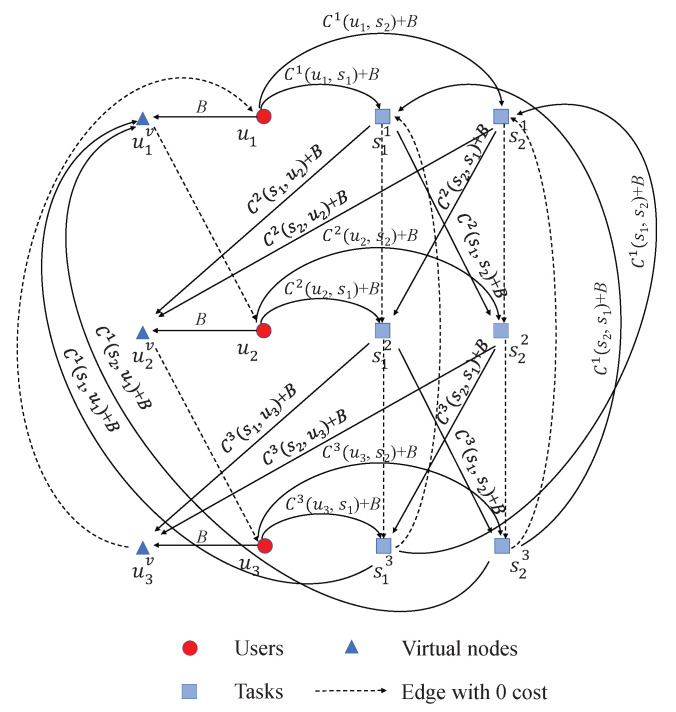
An example of the transformed graph of the single-TSP for 3 users and 2 task locations.

Here, *B* is a positive constant which is set to be $2\left(n+m\right)max_{i=1}^{n}max_{p,q=1}^{m}C^{i}\left(s_{p},s_{q}\right)$, and also large enough. If an edge does not have a cost in above equations, it does not exist in the transformed graph. Using the Equation (5), a transformed graph is obtained. Figure 4 demonstrates the transformed graph for 3 users and 2 task locations.

Then, we prove the equivalence of the transformed single-TSP and the initial multiple-TSP in the following theorem.

**Theorem** **1.** *Given an optimal solution, $y_{opt}$, of the single-TSP, the optimal solution of the multiple-TSP could be achieved in n+m steps, which is a set of tours $R_{1}$,...,$R_{n}$ [19].*

**Proof.**  We give a common assumption that the optimal solution $y_{opt}$ starts from the initial location of the first user, $u_{1}$. To prove the Theorem 1, we state the following lemmas:

**Lemma** **1.** *The optimal solution $y_{opt}$ for the single-TSP has some natures, which are listed as follows:*

1. *We define the virtual location set corresponding to task location $s_{j}$ as $L_{j}=\left\{s_{j}^{i}:i=1,\cdots ,n\right\}$. Moreover, we make user that there is only one edge that comes into and departs from $L_{j}$.*
2. *Assume that $s_{j}^{i}$ is the first virtual location in $L_{j}$ visited by the path in the optimal solution, after that, the path will visit all the remaining virtual locations in $L_{j}$ before leaving $L_{j}$.*
3. *The user route, $P_{i}$, from the initial location $u_{i}$ to its corresponding terminal point $u_{i}^{v}$ in $y_{opt}$ will not pass through any other users’ initial locations and terminal points.*
4. *The cost of the optimal solution $C(y_{opt})$ is equal to the summation of all the route costs of users, i.e., $\sum_{i=1}^{n}C\left(P_{i}\right)$.*

**Proof.**  The cost of the incoming and outgoing edges of $L_{j}$ would have a value *B* associated to it. If the user route in the optimal tour leaves $L_{j}$ without visiting all the virtual locations in $L_{j}$, there will be other paths entering $L_{j}$ to visit remaining locations whose cost is at least greater than *B*. Since the optimal solution would have a least number of edges whose costs are no less than *B*, the number of edges entering and leaving $L_{j}$ is as few as possible. So nature 1 and nature 2 are proved. Due to nature 2, the transformed graph is such that the user route from $u_{i}$ after visiting a subset of the virtual locations must visit $u_{i}^{v}$ in the end. In other words, the user route can pass through any other users’ initial locations and terminal points only if nature 2 is violated. Therefore, nature 3 is true. For each terminal point, there is only one outgoing edge and the cost is zero. So all these edges $\left\{\left(u_{1}^{v},u_{2}\right),\left(u_{2}^{v},u_{3}\right),\ldots ,\left(u_{n}^{v},u_{1}\right)\right\}$ must exist in the optimal solution and removing all these edges will leave *n* unconnected user routes $P_{1},P_{2},\ldots ,P_{n}$. Hence, $C\left(y_{opt}\right)=\sum_{i=1}^{n}C\left(P_{i}\right)$, nature 4 is proved.    □

**Lemma** **2.** *Given an optimal solution on the transformed graph, $y_{opt}$, a set of tours $R_{1}$,...,$R_{n}$ are available for the multiple-TSP and the cost of multiple-TSP $\sum_{i=1}^{n}C\_\left(R_{i}\right)=y_{opt}-\left(n+m\right)B$. The above tours could be achieved in n+m steps.*

**Proof.**  We denote $\beta_{i}$ as the number of the virtual location sets visited by $P_{i}$ in the optimal solution, that is, $\beta_{i}$ is equal to the number of tasks that user *i* performs. If $\beta_{i}>0$, we denote the virtual location sets visited by $P_{i}$ as $L_{i1},L_{i2},\cdots ,L_{i\beta_{i}}$. The path visits the sets in the order of $L_{i1},L_{i2},\cdots ,L_{i\beta_{i}}$. Let the tour of the $i^{th}$ user, $R_{i}$, constructed from $P_{i}$ be $\left\{u_{i},s_{i1},s_{i2},\cdots ,s_{i\beta_{i}},u_{i}\right\}$. Specifically, $s_{ij}$ is the physical location corresponding to $L_{ij}$ and $s_{ij}^{k}$ is the virtual location corresponding to user *k* in $L_{ij}$ for all $j\in \{1,\ldots ,\beta_{i}\}$. When *i* is equal to 1, the following equation is available.

(6) $$\begin{array}{cc} C(P_{i}) & =C\left(u_{1},s_{11}^{1}\right)+\sum_{h=1}^{\beta_{1}-1}C\left(s_{1h}^{n},s_{1(h+1)}^{1}\right)+C\left(s_{1\beta_{1}}^{n},u_{1}^{v}\right)\\ \; & =C^{1}\left(u_{1},s_{11}\right)+B+\sum_{h=1}^{\beta_{1}-1}\left(C^{1}\left(s_{1h},s_{1(h+1)}\right)+B\right)\\ \; & +C^{1}\left(s_{1\beta_{1}},u_{1}\right)+B\\ \; & =C\_\left(R_{1}\right)+\left(\beta_{1}+1\right)B. \end{array}$$

Without loss of generality, the equation is workable for any i>1. When $\beta_{i}=0$, $P_{i}$ is made up of only one edge $\left(u_{i},u_{i}^{v}\right)$, so $R_{i}=\emptyset$ and $C\_(R_{i})=0$ in this case. So the cost of the optimal solution, $C(y_{opt})$, can be represented as Equation (7). For any *i*, $R_{i}$ can be transformed from $P_{i}$ in $\beta_{i}+1$ steps. Hence, all the tours are available in n+m steps from $y_{opt}$.

(7) $$\begin{array}{cc} C(y_{opt}) & =\sum_{i=1}^{n}C\left(P_{i}\right)\\ \; & =\sum_{i=1,\beta_{i}>0}^{n}C\left(P_{i}\right)+\sum_{i=1,\beta_{i}=0}^{n}C\left(P_{i}\right)\\ \; & =\sum_{i=1}^{n}C\_\left(R_{i}\right)+\left(m+n\right)B. \end{array}$$

   □

**Lemma** **3.** There are some optimal tours, $R_{1}^{*},\cdots ,R_{n}^{*}$, of the multiple-TSP. A feasible solution y can be constructed on the transformed graph which satisfies $\sum_{i=1}^{n}C\_\left(R_{i}^{*}\right)=C\left(y\right)-\left(n+m\right)B$.

**Proof.**  If $R_{i}^{*}$ contains no point for user *i*, let $P_{i}$ consists of only one edge $\left(u_{i},u_{i}^{v}\right)$. Otherwise, assume that $R_{i}^{*}$ is represented by $\left\{u_{i},s_{i1},s_{i2},\cdots ,s_{i\beta_{i}},u_{i}\right\}$, we can build $P_{i}$ that starts from $u_{i}$ and visits all the virtual location sets in the order of $L_{i1},L_{i2},\cdots ,L_{i\beta_{i}}$ and arrives at the terminal point $u_{i}^{v}$. Then, add the zero cost between the terminals and initial locations ($i.e.,\;\{\left(u_{1}^{v},u_{2}\right),\left(u_{2}^{v},u_{3}\right),\ldots ,\left(u_{n}^{v},u_{1}\right)\}$). So a feasible solution *y* for single-TSP is available on the transformed graph. Considering Lemma 2 in the reverse method, the following equation: $\sum_{i=1}^{n}C\_\left(R_{i}^{*}\right)=C\left(y\right)-\left(n+m\right)B$ could be proved.    □

We can build the tours for the multiple-TSP as in Lemma 2. According to Lemmas 2 and 3, the following equation is available.

(8) $$\begin{array}{cc} \sum_{i=1}^{n}C\_\left(R_{i}\right) & =C\left(y_{opt}\right)-\left(n+m\right)B\\ \; & \leq C\left(y\right)-\left(n+m\right)B=\sum_{i=1}^{n}C\_\left(R_{i}^{*}\right) \end{array}$$

Hence, the tours, $\left\{R_{i}:i\in \left\{1,\cdots ,n\right\}\right\}$, is optimal for the multiple-TSP. Theorem 1 is proved.    □

### 3.3. Single-TSP Solution

#### 3.3.1. Greedy Algorithm

Since the minimal TSP in this paper does not satisfy the geometric nature, that is, the sum of two sides is larger than the third side in any triangle, it is difficult to find a greedy algorithm whose approximate performance satisfies bound [20], so we transform the minimization problem to maximization problem.

As shown in Algorithm 1, PTAM-Greedy works as follows, we first take the transformed graph G=(V,E,C(E)) obtained in the previous section as input, where *V* represents the node set, *E* is the edge set in *G* and C(E) denotes the cost function on the set of edges *E*. Then we calculate the maximum cost $C(e_{max})$ for all edges and redefine the cost of each edge as $C^{\prime }\left(e_{i}\right)=C\left(e_{max}\right)-C\left(e_{i}\right)$ and obtain the new graph $G^{\prime }$. Now we have transformed the asymmetric minimization TSP into the maximization TSP. Moreover, we run Algorithms 2 and 3 on the graph $G^{\prime }$ using the new cost function $C^{\prime }$, each algorithm returns a Hamiltonian tour and we take the heavier Hamiltonian tour of Algorithms 2 and 3 as the final solution [21]. In this way, we can obtain the guaranteed approximation performance of PTAM-Greedy as $\frac{8}{13}$. Next, we introduce Algorithms 2 and 3 in turn.
**Algorithm 1** PTAM-Greedy.Input: the transformed graph *G* = (*V*,*E*, C(E))Output: a Hamiltonian tour on *G*1:Let $C(e_{max})=$ max $\left\{C\left(e_{i}\right)\right\}$ for $\forall e_{i}\in E$.2:Define a new cost function $C^{\prime }\left(e_{i}\right)=C\left(e_{max}\right)-C\left(e_{i}\right)$ for $\forall e_{i}\in E$.3:Run Algorithms 2 and 3 on the graph $G^{\prime }=\left(V,E,C^{\prime }\left(E\right)\right)$ with new cost function, respectively.4:Return the heaviest tour as the final solution.

**Algorithm 2** GHT.
Input: a graph *G* = (*V*,*E*, C(E))
Output: a Hamiltonian tour on *G*
1:Compute a maximum weight cycle cover *Y* of *G* with greedy method.2:Define a new cost function $C^{\prime }$ for edges in *E*. $\forall \;i\in I_{2}$, $C^{\prime }\left(\left(s_{i},t_{i}\right)\right)=C^{\prime }\left(\left(t_{i},s_{i}\right)\right)=2\left(b_{i}-c_{i}\right)$.3:$\forall \;i,j\in I_{2}$, i≠j, $C^{\prime }\left(\left(t_{i},s_{j}\right)\right)=C\left(\left(t_{i},s_{j}\right)\right)+\left(b_{i}-c_{i}\right)+\left(b_{j}-c_{j}\right)$.4:$\forall \;i\in I_{2}$, if $u\notin \{t_{k}|k\in I_{2}\}$ and $v\notin \{s_{k}|k\in I_{2}\}$, $C^{\prime }\left(\left(u,s_{i}\right)\right)=C\left(\left(u,s_{i}\right)\right)+\left(b_{i}-c_{i}\right)$, at the same time, $C^{\prime }\left(\left(t_{i},v\right)\right)=C\left(\left(u,s_{i}\right)\right)+\left(b_{i}-c_{i}\right)$.5:For other edges *e*, $C\left(e\right)=C^{\prime }\left(e\right)$.6:Compute the maximum perfect matching *M* on $G=(V,E,C^{\prime }\left(E\right))$.7:Delete the edge with the smallest weight of each cycle in *Y* except 2-nodes-cycles and get a set of paths *P*.8:Let *T* denote the set of 2-nodes-cycles in *Y* which do not have the common edge with *M*.9:Let $\overset{ˇ}{M}$ denote all the edges in *M* but not in any 2-nodes-cycle.10:Form the graph $\tilde{G}=\left(V,T\cup P\cup \overset{ˇ}{M}\right)$ and color the edges in $\tilde{G}$ into two colors.11:For all 2-nodes-cycles out of *T*, add the edge with larger weight to the above two color sets. For each 2-nodes-cycle $Y_{i}$, $e_{m}$ and $e_{n}$ represent two edges that connect the nodes in $Y_{i}$, then color the edge of $Y_{i}$ adjacent to $e_{m}$ the same color as $e_{m}$ and the edge adjacent to $e_{n}$ the same color as $e_{n}$.12:Connect the paths in the color set with larger total weight randomly and get the solution.


**Algorithm 3** GHTCAN.
Input: a graph *G* = (*V*,*E*, C(E))
Output: a Hamiltonian tour on *G*
1:Compute a maximum weight cycle cover *Y* of *G* with greedy method.2:Delete the edge with smallest weight of each cycle and achieve a group of paths *P*.3:Connect all the paths in *P* arbitrarily and get the solution.


As for Algorithm 2, we first find the maximum cycle cover *Y* of the transformed graph *G* using the greedy method in line 1, where the cycle cover of graph *G* is a group of the node disjoint cycles which contains all nodes. Let $Y_{1}$, $Y_{2}$,..., $Y_{l}$ denote all cycles in cycle cover *Y*. In the greedy method, we search a cycle with the highest weight, then continue to search next cycle with the highest weight at the remaining nodes until getting the cycles which cover all nodes. In this paper, we denote $I_{k}$ as the set of all indices *i* such that $Y_{i}$ is a *k*-cycle. Then in line 2 we redefine the cost function for each edge in *E*, the changes mainly happen in 2-nodes-cycles, where $\left(s_{i},t_{i}\right)$ denotes the heavier edge in $Y_{i}$ for all $i\in I_{2}$, $b_{i}$ is the greater weight in $Y_{i}$ and $c_{i}$ is the lower one. Then in lines 3–4, we add the weight of $b_{i}-c_{i}$ to all the edges adjacent to the edge $\left(s_{i},t_{i}\right)$ with larger weight. Moreover, we calculate a maximum perfect matching *M* in line 6, the perfect matching is a set of edges without common nodes, which covers all nodes in *V*. Then in lines 7–9, we delete the edge with smallest weight for each cycle which covers at least 3 nodes and get a group of node disjoint paths *P*. Next, in lines 10–11 we compress the 2-nodes-cycles into a single node and obtain the graph $\tilde{G}$, then according to the coloring lemma [21], we color all edges in graph $\tilde{G}$ into two colors such that the edges in each color set could form a nodes disjoint path set. Finally, we connect the paths of the color set with heavier total weight arbitrarily and get a Hamiltonian tour.

**Figure 5:**
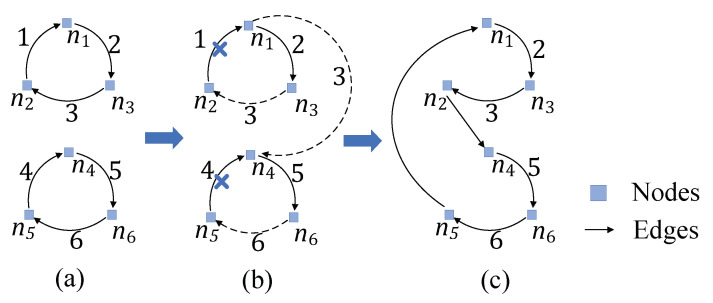
A simple execution process of the Algorithm 2. (**a**) initial state. (**b**) computing M. (**c**) find maximum cycle.

For example, assuming that there are 6 nodes $n_{1},\ldots ,n_{6}$ in graph *G*, we first compute the maximum weight cycle cover *Y* of *G*. In Figure 5a, $\left(n_{1},n_{3},n_{2}\right)$ and $\left(n_{4},n_{6},n_{5}\right)$ are the all two 3-nodes-cycles in the cycle cover *Y* that we have found with the greedy method, and the number next to the edge represents the weight in Figure 5a. Then we change the weight for each edge and compute the maximum perfect matching *M* according to Algorithm 2, afterwards, we remove the lightest edge in each 3-nodes-cycle. As Figure 5b shows, all the edges in $M=\{\left(n_{3},n_{2}\right),\left(n_{6},n_{5}\right),\left(n_{1},n_{4}\right)\}$ are drawn dashed, meanwhile, $\left(n_{2},n_{1}\right)$ and $\left(n_{5},n_{4}\right)$ are deleted as the lightest edge in each 3-nodes-cycle. Moreover, according to the coloring lemma [21], we color all the edges into two colors and select the edges $\left(n_{1},n_{3}\right)$, $\left(n_{3},n_{2}\right)$, $\left(n_{4},n_{6}\right)$ and $\left(n_{6},n_{5}\right)$ in the color collection with heavier total weight as shown in Figure 5c. Finally, we connect these edges arbitrarily, in this example, there is only one feasible connection method, thus we connect node $n_{2}$ to $n_{4}$ and node $n_{5}$ to node $n_{1}$ and get the final Hamiltonian tour.

In Algorithm 3, similar to Algorithm 2, we first find the maximum cycle cover *Y* of the transformed graph *G*. Then in line 2 we remove the edge with lightest weight for each cycle in *Y* and achieve a group of node disjoint paths *P*. Finally, we connect all paths in *P* arbitrarily and get a Hamiltonian tour which covers all nodes in *G* as the final solution.

**Theorem** **2.** *Using Algorithm 1 to solve the minimal TSP could achieve the performance bound of $\frac{5}{13}\frac{C(e_{max})}{C(e_{min})}+\frac{8}{13}$ to the optimal solution.*

**Proof.**  As described before, we we can obtain the guaranteed approximation performance of PTAM-Greedy as $\frac{8}{13}$ [21] through solving the maximal TSP by Algorithm 1. Then we give the following proving process.

First, we find the maximum cost edge $C(e_{max})$ and the minimum cost edge $C(e_{min})>0$ in the transformed graph, then the corresponding cost is changed from $C(e_{i})$ to $C\left(e_{max}\right)-C\left(e_{i}\right)$. Then, we record the optimal cost for minimization problem is $C_{opt}$, and the actual cost for minimization problem is $C_{PTAM}$. According to the bound of solving the above maximization problem, then we have:

(9) $$\begin{array}{c} \varphi C(e_{max})-C_{PTAM}\geq \frac{8}{13}\left(\varphi C(e_{max})-C_{opt}\right), \end{array}$$

where φ is the number of edges in a solution, and obviously, $\varphi C(e_{max})\leq \frac{C(e_{max})}{C(e_{min})}C_{opt}$, so we have:  

(10) $$\begin{array}{c} C_{PTAM}\leq (\frac{5}{13}\frac{C(e_{max})}{C(e_{min})}+\frac{8}{13})C_{opt}, \end{array}$$

Hence, for the minimization problem, through the above algorithm, we could get a bound of $\frac{5}{13}\frac{C(e_{max})}{C(e_{min})}+\frac{8}{13}$ to the optimal solution.    □

#### 3.3.2. Genetic Algorithm

**Figure 6:**
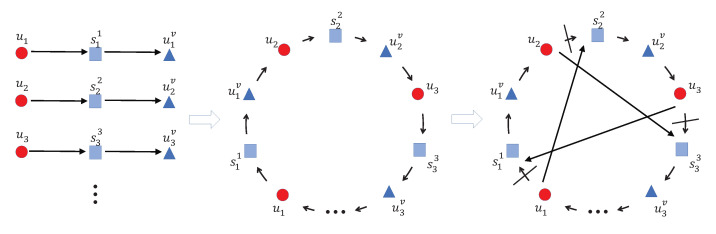
An example of fast-3-Opt heuristic.

With the purpose of further enhancing the performance of solving the above single-TSP, the genetic algorithm called PTAM-Genetic (as shown in Algorithm 4) is proposed starting with creating *p* individuals for the initial population by Nearest-Neighbor heuristic [22]. Then we adopt the fast-3-Opt heuristic [23] to transform the initial population into local optimal result as shown in Figure 6. The reason why we can’t use Lin-Kernighan heuristic [24] is because it adopts 2-opt moves which will change the direction of tours so that the tour length could be unpredictable. While the fast-3-Opt chooses a fragment and reinserts it into another position without changing direction of tours so that the algorithm could be used in solving the asymmetrical TSP.
**Algorithm 4** PTAM-Genetic Algorithm.1:Creates population *P* with Nearest-Neighbor heuristic;2:**for all** individual g∈P **do**3:    fast-3-Opt(*g*).4:**end for**5:**repeat**6:    **for** g=0 to #crossovers **do**7:        select two parents $g_{a},g_{b}\in P$ stochastically.8:        $g_{c}:=$ PTAMG-crossover $\left(g_{a},g_{b}\right)$.9:        fast-3-Opt($g_{c}$).10:        with predefined probability do PTAMG-mutation($g_{c}$).11:        replace an individual of *P* by $g_{c}$.12:    **end for**13:**until** converged.

After that, the PTAM-Genetic starts operating on its population by random choosing two individuals of the inputs to the crossover procedure. Then a crossover procedure called PTAMG-crossover, as shown in Algorithm 5, is employed.
**Algorithm 5** PTAMG-crossover ($g_{a},g_{b}$).1:$g_{c}:=g_{a}$.2:Remove all edges in $g_{c}$ that are not in $g_{b}$.3:Greedy_reconnect($g_{c}$).

In Algorithm 5, the contents of the first parent $g_{a}$ are copied to a new individual $g_{c}$. Then in line 2, the edges in $g_{c}$ that are not in $g_{b}$ are deleted, so $g_{c}$ contains a series of unconnected node sequence called fragment. Afterwards, as shown in line 3, a greedy reconnection operation is conducted on individual $g_{c}$ in function Greedy_reconnect. The detailed process is described as follows. Assume that there is a fragment (a,b) in $g_{c}$ where *a* is the start point and *b* is the endpoint. For each of other fragments, we can only connect the endpoint of the fragment to *a* or connect the start point of the fragment to *b*. Let $F_{a}$ denote the set of the fragments, for each fragment *f* in $F_{a}$, the edge between the endpoint of *f* and *a* exists in neither parent $g_{a}$ nor parent $g_{b}$. $f_{e}$ represents the fragment in $F_{a}$ whose endpoint can connect to *a* with minimum cost. While $F_{b}$ is denoted as the set of the fragments, for each fragment *f* in $F_{b}$, the edge between the start point of *f* and *b* exists in neither $g_{a}$ nor $g_{b}$. $f_{s}$ represents the fragment in $F_{b}$ whose start point can connect to *b* with minimum cost. Then, we select a fragment from $f_{e}$ and $f_{s}$ which can connect to (a,b) with minimum cost and connect it to (a,b). The process continues until all fragments are reconnected.

**Figure 7:**
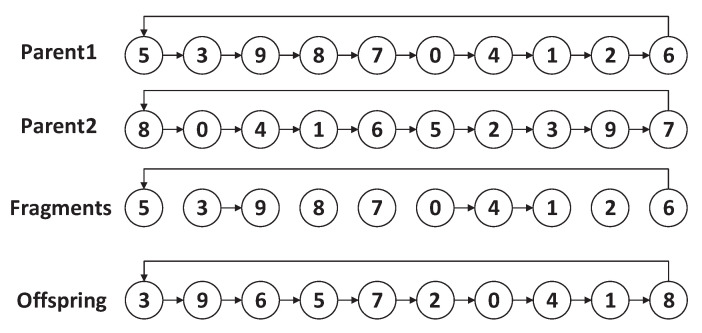
An example of crossover and greedy reconnection.

Let us give an example to explain the PTAMG-crossover. As shown in Figure 7, suppose that there are two parents, then we copy the first parent (Parent1) and delete all edges that do not exist in Parent2. As a result, we can get the fragments: (6, 5), (3, 9), (8), (7), (0, 4, 1), (2). Then, a fragment is chosen randomly as the start for the reconnection, for example, (3, 9). For the start point 3, the set $F_{a}$ contains {(8), (7), (0, 4, 1)} and the endpoint set is {8, 7, 1}.

For the endpoint 9, the set $F_{b}$ contains {(6, 5), (0, 4, 1), (2)} and the start point set is {6, 0, 2}. Assume that node 6 can connect to (3, 9) with the minimum cost among the endpoint set and the start point set, so node 6 is connected to node 9 and the fragment after reconnection is (3, 9, 6, 5). Through repeating the above process, all fragments are reconnected and the offspring is available in the end.

After finishing the crossover operation, the fast-3-Opt heuristic is employed to transform the offspring into a local best one. Then, the mutation as shown in Algorithm 6, is applied. It starts with stochastically deleting *k* edges from the individual (4≤k≤7, where *k* is randomly chosen, while if the total number of tasks in TSP is less than 14, then *k* is randomly chosen from 1 to half the number of tasks), and a greedy reconnection operation which is similar to the one for the crossover procedure is employed to reconnect the nearest while having not reconnected fragment. Finally, the mutated individual is handled with the fast-3-Opt to gain a local minimum.
**Algorithm 6** PTAMG-mutation (*g*).1:Randomly Choose *k* in an interval, which is determined by the total number of tasks.2:Remove *k* randomly chosen edges from *g*.3:Greedy_reconnect(*g*).4:fast-3-Opt(*g*).

The replacement strategy is important for maintaining adequate diversity within the population, which may also avoid premature convergence of the PTAM-Genetic algorithm. The replacement strategy proposed in this paper is described as follows. First, we consider the most similar (for the total cost performance) individual of the current population to the offspring. If the difference between them is lower than the predefined threshold, the individual should be replaced by the new offspring. While there is a special case, if the individual is the best one at present, then the individual will be replaced only when the new offspring has a lower total cost. If the new offspring has a larger total cost, then the individual with the largest total cost, while not the most similar individual in the current population, will be replaced by the new offspring.

## 4. Performance Evaluation

### 4.1. The Traces Used

**Figure 8:**
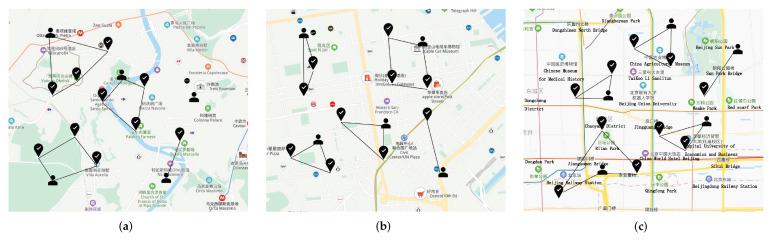
Performance comparisons on the three real-world data sets. (**a**) roma/taxi trace set. (**b**) epfl trace set. (**c**) geolife trace set.

Three data sets: roma/taxi trace set [25], epfl trace set [26], and geolife trace set [27,28] are adopted to test the performances of the task allocation strategies. The roma/taxi trace set includes 320 taxi drivers that work in the center of Rome, Italy. The epfl trace set contains mobility traces of taxi cabs in San Francisco, USA. While the geolife trace set contains 17,621 trajectories. We set the initial position as the points of users’ departures, and randomly select some positions (famous malls or views) as the task locations (as shown in Figure 8).

### 4.2. Algorithms in Comparison

To demonstrate the performance of the proposed task allocation strategies, we evaluate simulations of the following three aspects: (1) performances of PTAM-Greedy and PTAM-Genetic; (2) bound performance for the greedy algorithm; and (3) genetic algorithm’s performance along with the change of the number of generation. We take vast amounts of data by the simulations, while we consider the total cost performance, which is defined as the total cost consumed for users to complete all the tasks.

Three task allocation strategies: PTAM-Greedy, PTAM-Genetic and Random are compared to test the proposed algorithms. The first two strategies are proposed in this paper, while Random randomly assigns tasks to the users. In this paper, we consider task allocation process as a dynamic combinational optimization problem, while most methods regarded task allocation as a static allocation problem. In the dynamic special scenario, through a large number of literature review, such as [11,17,29], we found that most methods are improved on the basis of random. Therefore, we believe that the random method is widely representative in this scenario, and used the random method as the comparison method for experimental comparison.

### 4.3. Simulation Results

**Figure 9:**
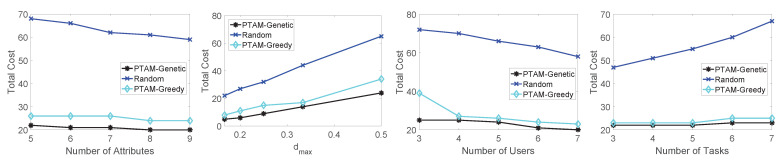
Performance comparisons on the roma/taxi trace set.

**Figure 10:**
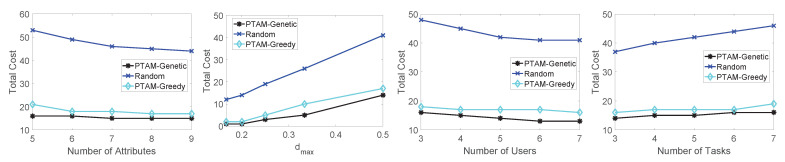
Performance comparisons on the epfl trace set.

**Figure 11:**
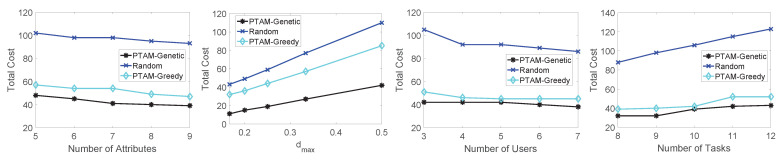
Performance comparisons on the geolife trace set.

In this section, we aim to evaluate the performance of the proposed algorithm. Specifically, we test the total cost along with the changing of the number of attributes, $d_{max}$, the number of users and the number of tasks. The simulation results on three different real-world data sets are illustrated in Figure 9, Figure 10 and Figure 11. In addition, The results of PTAM-Greedy are compared with the optimal results, meanwhile, the influence of PTAM-Genetic’s generation numbers to the total cost and execution times is tested. Finally, we compare the optimal results with the three algorithms along with the change of number of tasks on three data sets.

Firstly, we evaluate the performances of the three algorithm: PTAM-Genetic, PTAM-Greedy and Random on the roma/taxi trace set. As illustrated in Figure 9, we investigate the influence of the four variables to the total cost in different algorithms. Obviously, PTAM-Genetic consumes the lowest total costs in all four situations, while the performance of Random algorithm is the worst. The performance of PTAM-Greedy is far better than that of Random algorithm and close to that of PTAM-Genetic. Specifically, along with the increase of the number of attributes, the total cost of the three algorithms decreases slightly. The total costs of these algorithms increases along with the increase of the value of $d_{max}$, and $d_{iq}$ increases as the value of $d_{max}$ goes up. $d_{iq}$ represents the discount for $u_{i}$ to task $s_{q}$. When $d_{iq}$ increases, the cost will also increase. Furthermore, along with the growth of the number of users, the total cost will decrease. The reason is that when the number of users performing tasks increases, there will be more chances for a task to be assigned to an appropriate user, so the total cost is reduced. For changing the number of tasks, the performances of PTAM-Genetic and PTAM-Greedy are both far better than that of Random algorithm. The cost of PTAM-Greedy approximates to the cost of PTAM-Genetic but is slightly higher than that of PTAM-Genetic.

Secondly, in Figure 10, we compare the performances of the algorithms on epfl trace set. The simulation results show that the total cost performances rank as follows: PTAM-Genetic < PTAM-Greedy < Random, along with the change of the number of attributes, the value of $d_{max}$, the number of users and the number of tasks. The simulation results are reasonable and match the theoretical analysis. The total cost of PTAM-Genetic algorithm slightly decreases along with the change of number of attributes. The similar shapes also appear for PTAM-Greedy and Random algorithm. The total cost appears to be an upward trend for all three algorithms along with the growth of the value of $d_{max}$. With the increase of the number of users, the total costs of these algorithms decrease gradually. Moreover, The total cost slightly increases as the number of tasks goes up.

Thirdly, as shown in Figure 11, the performances of the algorithms are tested on geolife trace set. The total cost performance is still PTAM-Genetic < PTAM-Greedy < Random which is similar to the previous simulations.

Then, when number of users is 3 and number of tasks is 5, we conduct some simulations and get the results of PTAM-Greedy and the optimal results as shown in Table 2, where $C(e_{min})$ and $C(e_{max})$ denote the minimum and maximum cost of all edges, Proportion represents the ratio of the results of PTAM-Greedy and the optimal results, and Bound is the value calculated in Equation (10). In Table 2, we can find that as $C(e_{max})$ increases, the values of proportion fluctuate somewhat because the values of $C(e_{max})$ for all experiments are relatively close. However, the proportion is always less than the bound calculated by Equation (10) in each experiment, which means that simulation results match the theoretical analysis.

**Figure 12:**
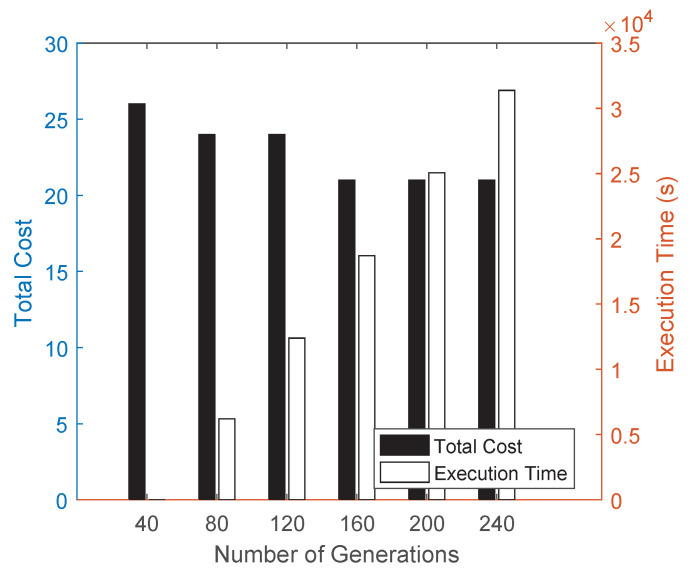
Performances along with a change of the number of generations.

Next, as shown in Figure 12, along with the number of generation changing from 40 to 240, we test total cost and execution time of roma/taxi trace set. It is not difficult to find that, the total cost of PTAM-Genetic algorithm is getting less, because along with generation growing, PTAM-Genetic algorithm can get a chance to find a better tour, so that the total cost will be lower. In addition, we can also see that the execution time of PTAM-Genetic algorithm is getting longer, because as generation grows, PTAM-Genetic algorithm takes time to find a better tour, so that the total execution time will get longer.

**Figure 13:**
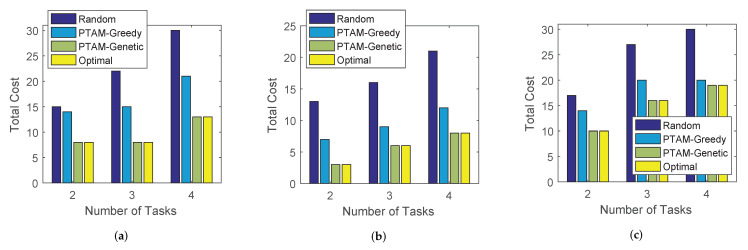
Performance comparisons on the three real-world data sets. (**a**) roma/taxi trace set. (**b**) epfl trace set. (**c**) geolife trace set.

Finally, as shown in Figure 13, the total cost of PTAM-Greedy, PTAM-Genetic, Random algorithm and the optimal results are compared along with the change of the number of tasks on three data sets. It is not difficult to find that the total cost performances rank as follows: Optimal = PTAM-Genetic < PTAM-Greedy < Random on all three real-world data sets. Due to the reason that the number of users in this simulation is set to 3 and the number of tasks varies from 2 to 4, the solution space is small. Therefore, the total costs of PTAM-Genetic are identical to that of the optimal results. Moreover, the total costs of all four algorithms increase with the growth of number of tasks in most cases.

## 5. Related Work

There are some works focusing on task allocations. Wang et al. [11] consider the heterogeneous user mobility model and dynamic arrivals of tasks, and present the offline combinatorial algorithm, then they mainly propose an online scheduling strategy based on the Lyapunov optimization with perturbation parameters to settle the problems in the new environment. Different from other studies that always focus on the task organizers, Wang et al. [30] mainly consider the attributes of participants such as user work bandwidth and mobility model, then they further consider the heterogeneity of tasks and participants and propose a novel task assignment framework. Guo et al. [31] focus on the worker selection problem in multi-task context, they consider both time-sensitive tasks and delay-tolerant tasks, and minimize the total distance and total number of selected workers, respectively. Then they present two genetic algorithms to settle the two optimization problems. In order to reduce energy consumption of vehicles and protect environment, Ding et al. [32] propose a cost-efficient path planning framework, which consists of two parts. One part is the cost consumption model considering the attributes of drivers and practical routes, the other is the real-time data collection with crowdsensing approach and path recommendation. Zhao et al. [33] consider task alloction from the perspective of task performers, and present a privacy-preserving unknow worker recruitment algorithm in crowdsensing, which is used to recruit the best workers to complete tasks without knowing the qualities of them completing tasks. They present a Differentially Private Multi-Armed Bandit game to model the unknown worker recruitment, and task completion quality contributed by each worker.

There are also some works taking the personalized problem into consideration. Yang et al. [34] study the fine-grained personalized task assignment considering users’ preferences and reliability level, then they present a task recommendation system to recommend tasks to users which consists of two parts, the first part is the method to quantify users’ preferences, the other one is the method to confer users’ reliability. In order to protect the privacy of users from being exposed when the server is hacked or under attacked, Wang et al. [35] present a distributed agent-based privacy-preserving framework, which uploads anonymous user information to a randomly selected agent at each upload to avoid exposing user trajectories to the proxy. They then locally perturb the crowdsourced data aggregated by each agent using Laplacian perturbation, and further combine the perturbed data from all agents for publication. An et al. [36] uses blockchain instead of data trading broker to record data transactions in Crowdsensed Data Trading, ensuring data truthfulness while protecting user privacy, and incentivizing consumers to rate truthfully the reliabilities of sellers. Wang et al. [37] consider the privacy protection of users’ locations and present a privacy-preserving task allocation framework, where users upload the ambiguous distances and locations rather than real ones, then they propose the winner selection strategy to select the users with ambiguous information and the payment determination strategy to ensure the truthfulness. Different from prior efforts, Jiang et al. [38] consider the similar sensing task data requirements for different workers as well as the heterogeneous attributes of workers and present a data-centric framework, which analyzes the common data in different tasks and reuses the common data to make full use of sensing resources and reduce the social costs. They also consider the private data of users and tasks and present a randomized auction strategy to maximize the social welfare. Lu et al. [39] use game theory to solve user’s inactive participation in multi-service exchange in MCS. They model the multi-service exchange problem as a Stackelberg multi-service exchange game consisting of multiple leaders and multiple followers, and present two novel algorithms to compute the unique Nash equilibrium for the sensing plan determination game and the reward declaration determination game, respectively. The only Stackerberg Equilibrium of the game is formed by these two algorithms together. Karaliopoulos et al. [40] study how to assign tasks to users and stimulate users efficiently with a novel view on payment distribution. They first obtain users’ preferences from historical data and formulate the optimization problem as a non-linear model, and finally they verify their mechanism by questionnaire. However, the above works usually regard task allocation as a static matching problem instead of a dynamic combinational optimization problem.

## 6. Conclusions

We have investigated the problem of task allocation in MCS campaigns through solving a combinatorial optimization problem. First, we propose a measurement method to calculate the cost for a user to complete a sensing task, taking both the distance and user’s preference into consideration. Then, we formulate the cost minimization problem as a heterogeneous, asymmetric, multiple-TSP. Through transforming multiple-TSP to single-TSP, we propose two algorithms to solve the multiple-TSP: greedy algorithm, which is proved to have a bound to the optimal solution, and genetic algorithm mixed with heuristic, which spends more calculation time while achieving a lower total cost. Finally, we have conducted a number of simulations based on three widely-used real-world traces: roma/taxi, epfl, and geolife. The simulation results could match the results of theoretical analysis.

## Figures and Tables

**Table 1:** List of key notations.

Notation	Description
U,S,A	the set of users, the set of tasks, the set of users’ preferences
$A_{u_{i}},A_{s_{i}}$	the preferences of user *i*, the preferences of task location $s_{i}$ that could satisfy some preferences of users
$u_{i},u_{i}^{v}$	the initial location of the user *i*, the terminal point of the user *i* on the transformed graph
$s_{j}^{i}$	the *j*-th virtual task location of user *i*
m,n	the number of task locations, the number of users
$C^{i}\left(u_{i},s_{i}\right)$	the cost of user *i* from $u_{i}$ to task $s_{i}$
$C^{i}\left(s_{p},s_{q}\right)$	the cost of user *i* from $s_{p}$ to $s_{q}$
$D(u_{i},s_{i})$	the physical distance between $u_{i}$ and $s_{i}$
$D(s_{p},s_{q})$	the physical distance between $s_{p}$ and $s_{q}$
$x_{iq}$	the $u_{i}$’s preference level for task $s_{q}$
$d_{iq}$	the discount for $u_{i}$ to task $s_{q}$
$P_{i}$	the path of user *i* in the transformed graph from the initial location $u_{i}$ to its corresponding terminal point $u_{i}^{v}$ in the optimal solution
$R_{i}$	the tour of user *i* in multiple-TSP
*G*	a transformed graph
*V*	the collection of nodes in graph *G*
*E*	the collection of edges in graph *G*
*Y*	a cycle cover in graph *G*
$Y_{1},\ldots Y_{l}$	the cycles in cycle cover *Y*
$I_{k}$	the set of all indices *i*, such that $Y_{i}$ is a *k*-vertices-cycle (k≥2)

**Table 2:** Results under the condition that 3 users, 5 tasks, $C_{min}=10$.

Parameter	Results			
	PTAM-Greedy	Optimal	Proportion	Bound
$C(e_{max})=15$	62	62	1	1.19
$C(e_{max})=16$	68	63	1.08	1.23
$C(e_{max})=17$	70	64	1.06	1.26
$C(e_{max})=18$	74	66	1.15	1.30
$C(e_{max})=19$	77	74	1.04	1.34

## Data Availability

The data ”roma/taxi trace set” used in this study are openly available at http://crawdad.org/roma/taxi/20140717/, accessed on 16 February 2022. The data ”epfl trace set” used in this study are openly available at http://crawdad.org/epfl/mobility/20090224/, accessed on 16 February 2022.
